# Extracellular Vesicles: Orchestrators of Intrahepatic and Systemic Crosstalk in Metabolic Dysfunction-Associated Steatotic Liver Disease

**DOI:** 10.3390/pharmaceutics18010116

**Published:** 2026-01-16

**Authors:** Yu Lei, Mei Liu, Xiang Tao

**Affiliations:** Department of Gastroenterology, Tongji Hospital, Tongji Medical College, Huazhong University of Science and Technology, Wuhan 430030, China

**Keywords:** extracellular vesicles, metabolic dysfunction-associated steatotic liver disease, diagnostics, treatment

## Abstract

Metabolic dysfunction-associated steatotic liver disease (MASLD) represents a multifaceted systemic condition, with the mechanisms linking intrahepatic lesions to systemic complications remaining a significant enigma in the field. This review posits that extracellular vesicles (EVs) serve as pivotal mediators facilitating communication between the liver and the entire organism. Within the hepatic environment, lipotoxic hepatocyte-derived EVs modulate macrophage populations and stellate cells, thereby promoting inflammatory and fibrotic processes. Systemically, the liver engages in bidirectional communication with adipose tissue, the intestinal tract, the cardiovascular system, and the pancreas via EVs, thus orchestrating metabolic homeostasis. Furthermore, we critically evaluate non-invasive diagnostic strategies and emerging therapies, including both natural and engineered EVs, based on EV-based interventions. We highlight the substantial potential and current challenges associated with achieving precision medicine in MASLD through targeted modulation of this specific communication network.

## 1. Introduction

Metabolic dysfunction-associated steatotic liver disease (MASLD), previously known as nonalcoholic fatty liver disease (NAFLD), has emerged as a complex multisystem signaling disorder, currently affecting approximately one-third of the global adult population. This condition transcends mere hepatic lipid accumulation, significantly disrupting inter-organ metabolic communication, with its prevalence rising in parallel with obesity and metabolic syndrome. In the absence of appropriate interventions, MASLD can advance to metabolic dysfunction-associated steatohepatitis (MASH), fibrosis, cirrhosis, and hepatocellular carcinoma (HCC) [1,2,3,4]. Although recent pharmacological treatments, such as resmetirom and semaglutide, aim at metabolic targets, a critical gap remains in our understanding of the mechanisms by which intrahepatic stress signals are systemically transmitted through specialized carriers, thereby contributing to disease progression [5,6,7].

Extracellular vesicles (EVs), lipid bilayer nanoparticles secreted by cells, have garnered increasing attention for their immunoregulatory roles in MASLD [8,9,10]. Notably, in addition to facilitating intrahepatic communication among various liver cell types, EVs serve as pivotal regulators of inter-organ crosstalk by transporting bioactive cargo capable of reprogramming the microenvironments of distant tissues [11,12,13,14], a mechanism that remains underexplored in the pathogenesis of MASLD. In the context of MASLD, the capacity of EVs to mirror the physiological state of their parent cells positions them as promising candidates for non-invasive biomarkers, providing valuable insights into disease progression and therapeutic responses [15,16,17]. Beyond their diagnostic potential, EVs are also being explored as therapeutic targets or agents in MASLD [18,19,20]. This review is distinguished from prior literature by its systematic characterization of MASLD as a systemic communication network disorder, mediated by EVs with a central focus on the liver. In this study, we not only synthesize the most recent findings on the role of EVs in interorgan communication but also critically assess their potential translational applications, ranging from biomarkers to engineered therapeutics. This work offers a comprehensive future roadmap for advancements in the field.

## 2. EVs Biogenesis and Cargo Loading

**Figure 1:**
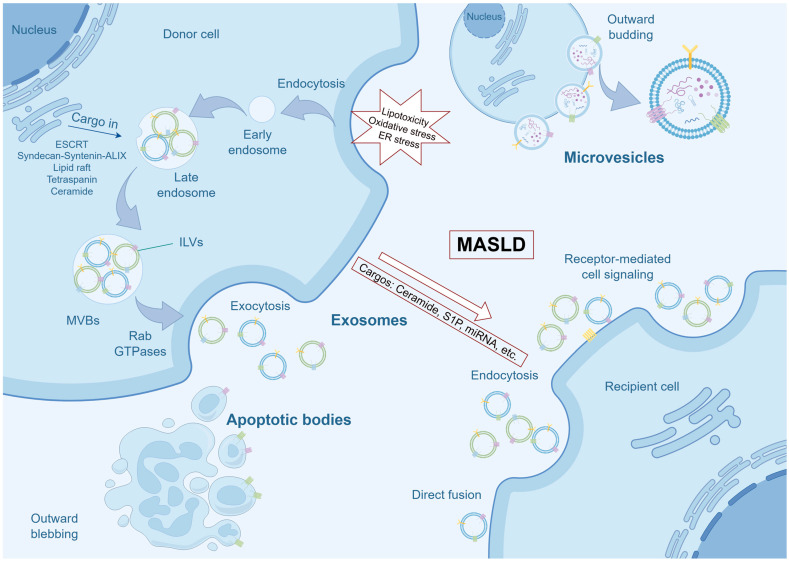
Biogenesis of EVs and Pathological Cargo Loading in MASLD. This schematic illustrates the primary pathways of EVs biogenesis and the pathological alteration of their cargo under MASLD conditions. In the context of MASLD, hepatocytes are subjected to lipotoxicity, oxidative stress, and ER stress. These pathological insults not only enhance the overall secretion of EVs but also critically reprogram their molecular cargo. Instead of random packaging, lipotoxic hepatocytes selectively load pathogenic molecules, such as ceramide, S1P, and specific miRNAs into the evolving EVs. Upon release, these pathologically charged EVs can mediate intercellular communication, thereby propagating inflammatory and pro-fibrotic signals that drive MASLD progression. ER, endoplasmic reticulum; ESCRT, Endosomal Sorting Complex Required for Transport; ILVs, intraluminal vesicles; MASLD, metabolic dysfunction-associated steatotic liver disease; miRNAs, microRNAs; MVBs, multivesicular bodies.

The biogenesis of EVs represents a complex cellular process characterized by distinct mechanisms [21] (Figure 1). Exosomes, a specific subset of EVs, are small vesicles (40–100 nm in diameter) originating from the endosomal system, particularly through the formation of multivesicular bodies (MVBs). The production of exosomes involves the inward budding of the endosomal membrane, resulting in the formation of intraluminal vesicles (ILVs) within MVBs. These MVBs can subsequently fuse with the plasma membrane, thereby releasing the ILVs as exosomes into the extracellular environment. The endosomal sorting complex required for transport (ESCRT) machinery is traditionally acknowledged for its pivotal role in the formation of ILVs within MVBs, serving as precursors to exosomes. The ESCRT machinery comprises several complexes, including ESCRT-0, -I, -II, and -III, which collectively mediate cargo selection, membrane budding, and vesicle scission in a sequential manner [22,23,24]. The ESCRT-0 complex initiates the endosomal sorting process by recognizing and clustering ubiquitinated cargo on endosomal membranes, thereby forming domains of aggregated cargo without inducing membrane deformation. This initial step is crucial as it establishes the foundation for subsequent membrane remodeling events. Following this, the ESCRT-I and ESCRT-II complexes collaborate to induce membrane deformation into buds, localizing at the bud necks and recruiting ESCRT-0-ubiquitin domains to these specific sites. This orchestrated action effectively confines the cargo within the budding vesicles [25]. The ESCRT-III complex is essential for the final scission of these buds, resulting in the formation of ILVs [26,27]. Moreover, the interaction between Syndecan, a heparan sulfate proteoglycan, and Syntenin, a cytoplasmic adaptor, with ALIX, an ESCRT accessory component, enhances the intraluminal budding of endosomal membranes, thus promoting exosome formation [28]. Additionally, ESCRT-independent pathways for exosome cargo sorting have been identified, encompassing mechanisms such as lipid raft, tetraspanin, and ceramide-mediated processes. These pathways offer alternative routes for cargo incorporation into exosomes, thereby enhancing the versatility of exosome-mediated communication [24,29,30,31,32]. Microvesicles, also known as ectosomes (50–1000 nm in diameter), are produced through the direct outward budding of the plasma membrane. This process is independent of the endosomal system and takes place at the cell surface, where the plasma membrane protrudes and subsequently pinches off to release microvesicles into the extracellular milieu [33,34]. Apoptotic bodies, larger EVs (800–5000 nm in diameter) generated during apoptosis, are encompassed within the spectrum of EVs, underscoring the complexity and functional diversity of these vesicles [35]. In addition, migrasomes introduce an additional layer of complexity to the EV landscape. These vesicles are formed on the retracting fibers of migrating cells and participate in a variety of physiological processes, such as embryogenesis, angiogenesis, and immune modulation [36].

EVs are critical mediators of intercellular communication, facilitating the transfer of proteins, lipids, and genetic material between cells. This process often necessitates docking at the plasma membrane, which can occur through mechanisms such as receptor binding, direct fusion, or endocytosis [34]. Understanding the molecular mechanisms that regulate EVs biogenesis and cargo loading is crucial for harnessing their full therapeutic potential. For instance, insights into EV biogenesis can be utilized in bioengineering applications to incorporate therapeutic proteins or nucleic acids into EVs, thereby enhancing their efficacy as drug delivery systems [37]. Additionally, the unique properties of EV membranes, such as enhanced cellular uptake and immune evasion, make them promising candidates for the development of biomimetic nanoparticles in gene therapy [38].

Importantly, in the pathological context of MASLD, characterized by lipotoxicity, oxidative stress, and endoplasmic reticulum (ER) stress, there is a notable increase in the secretion of EVs, accompanied by specific alterations in the sorting of their contents [39,40]. This indicates that pathogenic hepatocytes do not release EVs in a random manner; rather, they intentionally package pathogenic components such as ceramide, sphingosine-1-phosphate (S1P), and specific microRNAs (miRNAs) [11,41,42]. This pathological reprogramming underlies the transformation of EVs from mere cellular messengers to active promoters of disease, thereby facilitating subsequent intrahepatic and systemic pathological communication.

## 3. Intrahepatic EV-Mediated Cell Crosstalk in MASLD

### 3.1. Hepatocyte-Derived EVs

**Figure 2:**
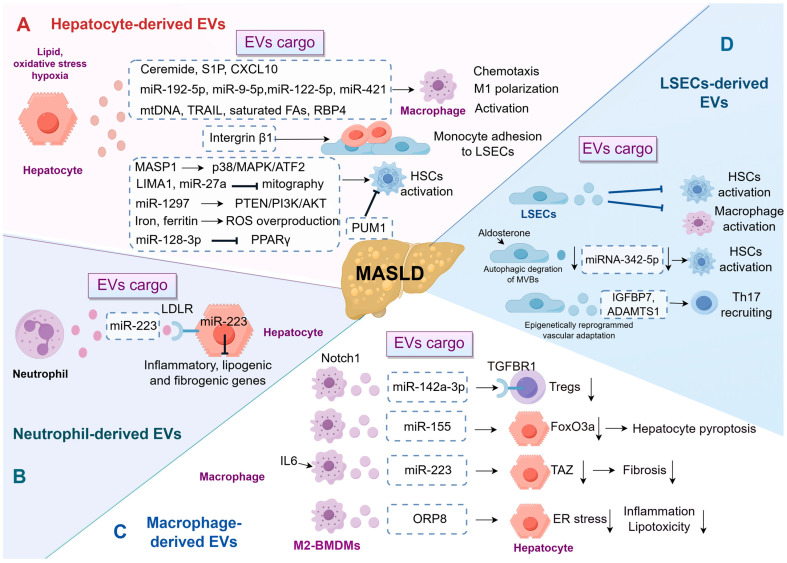
EV-Mediated Intrahepatic Immunoregulatory Network in MASLD. This schematic illustrates the intricate crosstalk among major hepatic cell types via EVs, which orchestrates inflammation and fibrosis in MASLD. (**A**) Hepatocyte-derived EVs: Lipotoxic hepatocytes secrete EVs enriched with diverse cargos that facilitate macrophage chemotaxis and M1 macrophage polarization, or activate HSCs, thereby promoting fibrogenesis. (**B**) Neutrophil-derived EVs: These EVs can deliver miR-223 to hepatocytes, inhibiting the expression of inflammatory and fibrogenic genes, thereby exerting a protective effect. (**C**) Macrophage-derived EVs: These EVs exhibit dual functions. On one hand, miR-155 from macrophages intensifies hepatocyte pyroptosis. Conversely, EVs from macrophages stimulated by IL-6, containing miR-223, can attenuate hepatocyte inflammation and fibrosis by downregulating targets such as TAZ. (**D**) LSEC-derived EVs: Under pathological conditions, the cargo composition of LSEC-derived EVs alters, shifting their function from anti-inflammatory and anti-fibrotic roles in homeostasis to promoting HSC activation and Th17 cell recruitment, thereby exacerbating fibrosis. ADAMTS1, a disintegrin and metalloproteinase with thrombospondin motifs 1; BMDMs, bone marrow-derived macrophages; CXCL10, C-X-C motif ligand 10; ER, endoplasmic reticulum; EVs, extracellular vesicles; FAs, fatty acids; HSCs, hepatic stellate cells; IGFBP7, insulin-like growth factor-binding protein 7; IL6, interleukin 6; LDLR, low-density lipoprotein receptor; LIMA1, LIM domain and actin binding 1; LSECs, liver sinusoidal endothelial cells; MASLD, metabolic dysfunction-associated steatotic liver disease; MASP1, mannan-binding lectin serine protease 1; mtDNA, mitochondrial DNA; ORP8, oxysterol-binding protein-related protein 8; PUM1, pumilio1; RBP4, retinol-binding protein 4; ROS, reactive oxygen species; S1P, sphingosine-1-phosphate; TAZ, transcriptional co-activator with PDZ-binding motif; TGFBR1, transforming growth factor beta receptor 1; Th17, T-helper 17; TRAIL, tumor necrosis factor-related apoptosis-inducing ligand; Tregs, regulatory T cells.

Lipotoxic hepatocytes are a major source of pathological EVs, which act as an activation hub for macrophages and hepatic stellate cells (HSCs) by delivering specific lipids, miRNAs, and proteins. Excessive lipid accumulation is a critical characteristic of MASLD, which can result in heightened oxidative stress in hepatocytes, mitochondrial dysfunction, ER stress, among other effects, ultimately leading to hepatocyte injury and death [43]. Research has indicated that lipid, oxidative stress, and hypoxia stimulate hepatocytes to release inflammatory EVs [40,44,45,46]. These hepatocyte-derived EVs have been shown to significantly impact macrophage behavior, thereby contributing to the progression of MASLD [40]. For example, in a 2020 study using a mouse model of MASH, Dasgupta et al. demonstrated that the activation of endoplasmic reticulum to nucleus signaling 1 (IRE1A) promotes the release of EVs. Importantly, the clinical relevance of this pathway was supported by correlative evidence from human cohorts [41]. Hepatocyte-derived lipotoxic EVs, enriched with ceramide and its metabolite S1P, have been demonstrated to induce macrophage chemotaxis via the S1P1 receptor, suggesting a mechanism by which these EVs facilitate macrophage infiltration into the liver under lipotoxic stress conditions [11,44] (Figure 2). Similarly, activating mixed lineage kinase 3 (MLK3) triggers the release of C-X-C motif ligand 10 (CXCL10)-enriched EVs from lipotoxic hepatocytes, inducing macrophage chemotaxis in mouse MASH models [47]. Furthermore, in the context of MASLD, lipotoxic hepatocytes have been found to deliver miR-192-5p, miR-9-5p, miR-122-5p, miR-421 via EVs, promoting M1 polarization of macrophages and contributing to the inflammatory milieu that accelerates disease progression. Specifically, in patients with MASLD, serum levels of miR-192-5p were found to correlate positively with hepatic inflammatory activity and disease progression, providing important clinical association [10,12,48,49]. Additionally, exosomal mitochondrial DNA (mtDNA), tumor necrosis factor-related apoptosis-inducing ligand (TRAIL), saturated fatty acids, and retinol-binding protein 4 (RBP4) derived from hepatocytes have been shown to promote the activation of macrophages, thereby accelerating the progression of MASLD [40,45,50,51]. Integrin β1, another cargo of EVs from lipotoxic hepatocyte, mediated monocyte adhesion to liver sinusoidal endothelial cells (LSECs), which contributed to hepatic inflammation and injury in a mouse model of MASH [52].

Moreover, EVs from steatotic hepatocytes have been demonstrated to provoke pro-fibrotic responses in cultured stellate cells, indicating that EVs serve as operational communicators in the pathophysiology of MASLD-associated liver fibrosis [53]. One significant study highlights the role of β-arrestin1 in modulating the release of hepatocyte-derived EVs enriched with mannan-binding lectin serine protease 1 (MASP1). These EVs activate HSCs through the p38 MAPK/ATF2 signaling pathway, promoting liver fibrogenesis in experimental models. Notably, the translational significance of this pathway is highlighted by clinical observations, as evidenced by the markedly elevated expression of MASP1 in both serum and liver tissue of patients with liver fibrosis. This suggests a robust association between this extracellular vesicle-associated mediator and human fibrotic disease [54]. Similarly, lipotoxic hepatocyte-derived EVs containing LIM domain and actin binding 1 (LIMA1) or miR-27a crucially promote HSC activation by hindering mitophagy, vital for cellular balance and stress response. In the clinical context, these findings suggest that elevated levels of LIMA1 are present in both the serum and serum small EVs of patients with MASH when compared to healthy controls [55,56,57]. Further supporting this, another study identified that lipotoxic hepatocyte-derived exosomal miR-1297 promotes HSC activation through the PTEN/PI3K/AKT signaling pathway [58]. Other cargos derived from hepatocytes, such as miR-128-3p, iron, and ferritin, can also induce the activation of HSCs, leading to the deposition of extracellular matrix (ECM) components and fibrosis. Notably, EV-mediated transfer of iron and iron-related components gains clinical relevance given the well-established observation that hepatic iron homeostasis is impaired in human MASLD/MASH [59,60,61]. However, the regulation of HSCs by hepatocyte-derived exosomes is not limited to activation. The RNA-binding protein Pumilio1 (PUM1) has been shown to be enriched in hepatocyte-derived exosomes, where it inhibits HSC activation by suppressing tropomyosin-4 translation. Importantly, this potentially protective mechanism may be compromised in MASLD, as the expression of PUM1 has been observed to be decreased in both patient samples and experimental models of the disease. This suggests that the loss of a regulatory EV cargo could contribute to the progression of hepatic fibrosis [62]. These conflicting findings, such as the dual roles of hepatocyte-derived EVs, indicate that their functions are context-dependent and warrant further investigation. By understanding the specific pathways and mechanisms through which these EVs influence HSC activation, novel therapeutic strategies can be developed to mitigate liver fibrosis and improve outcomes for patients with MASLD.

### 3.2. Neutrophil-Derived EVs

Neutrophils have emerged as significant players in chronic inflammatory conditions, including MASLD and its more severe form, MASH. Recent research has elucidated the dual role of neutrophils in both exacerbating and resolving inflammation in hepatic diseases. Neutrophils are implicated in the pathogenesis of MASLD by promoting oxidative stress and inflammation, which are crucial in the progression from simple steatosis to MASH [63,64,65]. This is further corroborated by the formation of neutrophil extracellular traps (NETs) [66]. Additionally, neutrophils can also secrete EVs and interact with hepatocytes to establish a complex network of EVs. For instance, a 2021 study conducted by He et al. highlights the role of neutrophil-derived miR-223 in ameliorating MASH by inhibiting hepatic inflammatory and fibrogenic gene expression. The selective uptake of miR-223-enriched EVs by hepatocytes, mediated by the low-density lipoprotein receptor (LDLR) and apolipoprotein E (APOE), underscores a targeted therapeutic potential for MASH [67].

The existing body of literature presents a seemingly paradoxical perspective on neutrophils, highlighting their dual role as both contributors to tissue injury through NETosis and mediators of inflammation resolution via miR-223-containing EVs. This dual functionality implies a highly context-dependent role for neutrophils, potentially influenced by factors such as disease stage, microenvironmental conditions, or metabolic status. A significant gap in current knowledge lies in elucidating the regulatory mechanisms that dictate whether neutrophils predominantly release injurious NETs or protective EVs. Additionally, while the therapeutic potential of targeting neutrophil-derived EVs is promising, it remains in its early stages. Key challenges include the selective modulation of EV release in vivo, ensuring the stability and targeting efficiency of administered EV preparations, and mitigating potential off-target effects. Future research should prioritize validation in human cohorts, employ single-cell analyses to distinguish neutrophil subpopulations in MASLD, and conduct mechanistic studies to uncover the upstream signals that regulate their EV secretory phenotype.

### 3.3. Macrophage-Derived EVs

Macrophages, particularly liver-resident macrophages known as Kupffer cells (KCs), play a pivotal role in maintaining liver immunity and homeostasis. KCs are self-renewing and predominantly non-migratory, functioning as sentinels to preserve liver equilibrium. In response to hepatic injury, KCs become activated and secrete inflammatory cytokines and chemokines, which recruit monocytes that subsequently differentiate into monocyte-derived macrophages. This recruitment and differentiation process is essential for the liver’s injury response and subsequent repair mechanisms [68,69]. Macrophages undergo polarization towards a pro-inflammatory phenotype, thereby facilitating the disease’s progression from simple steatosis to MASH and fibrosis [70,71].

In hepatic macrophages, Notch1 signaling plays a crucial role in modulating regulatory T cells (Tregs) via the exosomal miR-142a-3p/TGFBR1 axis [9]. The modulation of Tregs by macrophage-derived EVs presents a potential therapeutic target for early intervention in MASLD, aiming to restore immune equilibrium and prevent disease progression. Beyond immune cells, the impact of macrophage-derived EVs on hepatocytes reveals a network of opposing signals. On one hand, certain EVs propagate injury. For instance, macrophage-derived exosomal miR-155 exacerbates hepatocyte pyroptosis and fibrosis by downregulating FoxO3a [72]. Conversely, other EV populations convey protective cues. For example, myeloid-specific interleukin-6 (IL-6) signaling promotes the production of miR-223-enriched exosomes from macrophages, which play a crucial role in mitigating liver fibrosis by downregulating the profibrotic transcriptional co-activator with PDZ-binding motif (TAZ) in hepatocytes. The clinical relevance of this regulatory axis is supported by human cohort studies [73]. The integrity of this beneficial communication can be compromised in MASLD, as palmitic acid (PA) treatment impairs the uptake of these protective miR-223-loaded EVs by downregulating hepatic LDLR expression [74]. Further reinforcing the protective axis, EVs from M2-polarized bone marrow-derived macrophages (BMDMs), enriched with oxysterol-binding protein-related protein 8 (ORP8), mitigate lipotoxicity and inflammation by alleviating ER stress in recipient cells [75]. This synthesis underscores that macrophage-derived EVs are not uniformly pathogenic. The therapeutic challenge and opportunity lie in selectively targeting deleterious EV signals while preserving or enhancing the beneficial ones, thereby shifting the intercellular communication balance toward restoration of hepatic health.

### 3.4. LSEC-Derived EVs

LSECs are recognized for their pivotal role in maintaining hepatic homeostasis and regulating immune responses. They engage in filtration, endocytosis, and antigen presentation, processes that are integral to the immune response during hepatic injury [76]. In the context of MASLD, LSECs undergo pathophysiological alterations characterized by capillarization, a process in which LSECs lose their fenestrations and adopt a more vascular phenotype. This transformation is significant as it precedes and potentially facilitates the progression of liver fibrosis and inflammation [77,78].

Recent investigations into LSECs within the context of MASLD and the role of EVs in this condition represent a rapidly expanding field of research. The ability of LSEC-derived EVs to modulate the activation states of HSCs and KCs suggests a protective mechanism against the fibrogenic and inflammatory processes associated with MASLD [79]. In contrast, another study highlights the role of autophagy in the degradation of MVBs within LSECs, leading to a decrease in both the quantity and quality of EVs, which in turn activates HSCs [80]. The maladaptive response of liver endothelial cells (ECs) to enhance the production of angiocrine factors such as insulin-like growth factor-binding protein 7 (IGFBP7) and a disintegrin and metalloproteinase with thrombospondin motifs 1 (ADAMTS1) within EVs is a pivotal mechanism in the recruitment of fibrogenic T-helper 17 (Th17) cells to the liver, intricately associated with the pathogenesis of liver fibrosis. The maladaptive EV-mediated vascular-immune axis, which has been mechanistically elucidated in animal models, has been substantiated in human cirrhotic liver through comprehensive multi-omics analyses, thereby underscoring its significance in the pathology of human disease [81]. These studies indicate that alterations in the status of LSECs in MASLD, such as capillarization, significantly modify the composition or function of their secreted EVs, shifting their role from anti-inflammatory and anti-fibrotic to pro-fibrotic.

This section provides an overview of the intricate communication occurring among intrahepatic cells via EVs. Nonetheless, the majority of the supporting evidence is derived from in vitro co-culture systems or murine models, and its applicability to human MASLD remains to be substantiated. The heterogeneity in EV isolation techniques across different studies complicates the direct comparison of cargo profiles. Furthermore, while various signaling pathways, such as S1P and miR-192-5p, have been identified, their relative significance at different stages of the disease, as well as the potential for synergistic interactions, remains to be elucidated.

## 4. Inter-Organ EV Signaling: MASLD as a Systemic Disease

MASLD is increasingly recognized as a systemic disease with significant extrahepatic manifestations. This recognition stems from its association with a range of metabolic disorders, including cardiovascular disease (CVD), type 2 diabetes mellitus (T2DM), chronic kidney disease (CKD), and certain types of cancers. The systemic nature of MASLD is underscored by its pathophysiological mechanisms, which include insulin resistance (IR), oxidative stress, and inflammation, all of which contribute to its progression and the development of related comorbidities [2,82,83].

### 4.1. Liver–Adipose Axis

**Figure 3:**
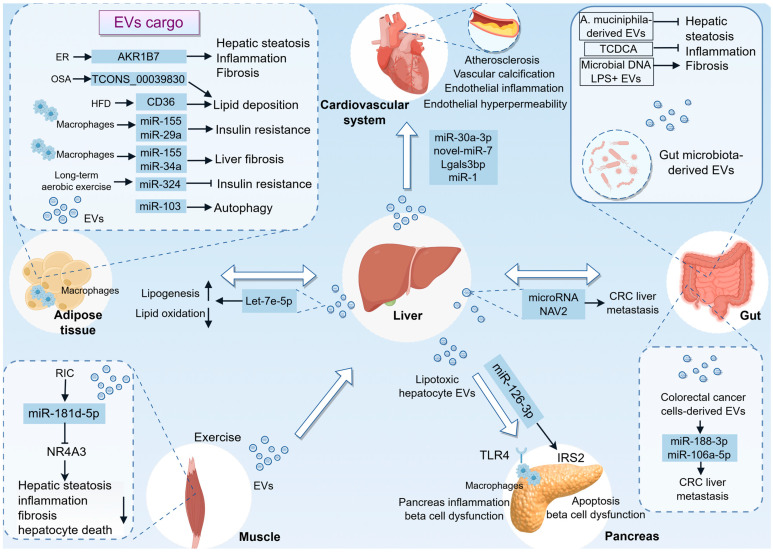
Systemic EV Trafficking in MASLD: A Multiorgan Communication Network. This figure summarizes the complex inter-organ crosstalk mediated by EVs in MASLD, positioning the diseased liver as a central hub in a systemic pathological network. (1) Liver–adipose axis: Adipose tissue-derived EVs have been shown to promote hepatic lipid accumulation, inflammation, and fibrosis. However, EVs released from adipose tissue following exercise, which contain miR-324, have beneficial effects. Additionally, the liver secretes EVs enriched with molecules such as let-7e-5p to modulate adipocyte function. (2) Liver–gut axis: The gut microbiota and their derived EVs play a significant role in influencing liver homeostasis, either exacerbating or ameliorating hepatic injury. CRC cell-derived EVs deliver miR-188-3p and miR-106a-5p, activating HSCs and inducing M2 macrophage polarization, respectively, thereby fostering a pre-metastatic niche conducive to liver metastasis. (3) Liver–cardiovascular axis: Hepatocyte-derived EVs contribute to endothelial inflammation, promote the osteogenic differentiation of vascular smooth muscle cells, and facilitate macrophage foam cell formation, thereby accelerating the processes of atherosclerosis and vascular calcification. (4) Liver–pancreas axis: EVs from hepatocytes can induce pancreatic inflammation through TLR4 signaling and cause β-cell apoptosis and dysfunction via miR-126a-3p. (5) Liver–muscle axis: Exercise or RIC stimulates the release of muscle-derived EVs enriched with specific miRNAs, which act on the liver to mitigate injury. AKR1B7, aldo-keto reductase 1B7; CRC, colorectal cancer; ER, endoplasmic reticulum; EVs, extracellular vesicles; HFD, high-fat diet; LPS+, lipopolysaccharide-positive; NAV2, neuron navigator 2; NR4A3, nuclear receptor 4A3; OSA, obstructive sleep apnoea; RIC, remote limb ischemic conditioning; TCDCA, taurochenodeoxycholic acid; TLR4, Toll-like receptor 4.

Inter-organ EVs signaling is integral to the pathophysiology of MASLD, a condition characterized by a spectrum of liver injuries. Adipose tissue serves as a significant source of circulating EVs, which are crucial in regulating gene expression within the liver [84]. Emerging evidence indicates that EVs originating from adipose tissue play a role in modulating insulin sensitivity in individuals with obesity and MASLD [85] (Figure 3). These EVs can impair signal transduction downstream of insulin receptors by transporting miRNAs, such as miR-155 and miR-29a [86,87]. In contrast, exosomal miR-324 derived from adipose tissue post-exercise appears to confer protection against IR and hepatic lipid accumulation, thereby providing insights into the molecular pathways influenced by aerobic exercise. This is supported by a clinical intervention study in which exosomes were isolated from MASLD patients after a 12-week aerobic exercise program [88]. These findings are significant for understanding the role of exercise-derived exosomes in promoting metabolic health, particularly through the regulation of Rho-associated protein kinase 1 (ROCK1), a known target of miR-324. Additionally, recent research has underscored the contribution of visceral adipose tissue (VAT)-derived EVs to the pathogenesis of MASH [89]. Mechanistically, the study conducted by Li et al. demonstrated that a high-fat diet (HFD) leads to the inhibition of AMPKα1 in adipocytes, resulting in an increased release of exosomes. This process facilitates the delivery of CD36 to hepatocytes, thereby promoting lipid accumulation and inflammation [90]. Specifically, adipocyte-derived exosomes from obstructive sleep apnoea (OSA) rats have been shown to aggravate MASLD by modulating the TCONS_00039830/miR-455-3p/Smad2 axis, which plays a crucial role in lipid metabolism and inflammation [91]. In this context, the role of ER stress in adipocytes is crucial, as it triggers the secretion of EVs containing aldo-keto reductase 1B7 (AKR1B7). This process results in elevated glycerol levels in hepatocytes, which in turn exacerbates lipid accumulation and inflammation, ultimately contributing to the development of MASH [92]. Another study found that miR-103, present in adipose-derived exosomes, can exacerbate MASH by modulating autophagy-related pathways [93]. These mechanisms are critical as they establish a link between peripheral adipose tissue dysfunction and hepatic lipid metabolism and inflammation, both of which are central to the progression of MASH. More recently, miR-155 and miR-34a have been identified as key fibrogenic miRNAs within small EVs secreted by adipose tissue macrophages (ATMs) in MASH, highlighting their mechanistic significance in the progression of liver fibrosis associated with obesity. Co-treatment with antagomirs targeting these miRNAs effectively inhibited the fibrogenic effects of these EVs both in vitro and in vivo, suggesting a promising strategy for therapeutic intervention [94].

In turn, liver can also secrete EVs that target adipocytes, thereby regulating adipogenesis and lipogenesis in response to lipid overload. Research has demonstrated that lipid overload upregulates the expression of geranylgeranyl diphosphate synthase (Ggpps) in the liver. This upregulation subsequently influences the secretion of miRNA-loaded EVs through the geranylgeranylation of Rab27A. Among these miRNAs, let-7e-5p has been implicated in promoting lipid accumulation within adipocytes, indicating a direct connection between liver-derived signals and adipocyte function. It is noteworthy that the functional role of let-7e-5p in promoting lipid accumulation has been predominantly demonstrated in experimental models. In contrast, the significance of this axis in humans is corroborated by observational data, which reveal correlations between plasma EV-derived let-7e-5p levels, hepatic Ggpps expression, and metabolic parameters such as body mass index (BMI) and liver triglyceride content in patients with MASLD [13]. This finding highlights the liver’s capacity to detect metabolic states and transmit appropriate signals to adipose tissue, thereby facilitating an adaptive response to lipid overload.

### 4.2. Liver–Gut Axis

The complex interaction between the gut and liver is increasingly acknowledged as a pivotal factor in the development of MASLD. By focusing on the gut–liver axis, it is feasible to devise more effective strategies for the prevention and treatment of MASLD, thereby enhancing patient outcomes and alleviating the burden of this increasingly prevalent condition [95,96,97,98]. Recent investigations into gut-derived EVs within the context of MASLD have attracted considerable attention due to their potential role in facilitating gut–liver interactions [99,100,101]. Specifically, gut-derived EVs, particularly those containing microbial DNA, have been implicated in the modulation of inflammatory responses and metabolic processes that are central to the pathogenesis of MASLD [102,103]. Furthermore, research indicates that EVs derived from fecal matter, especially those from patients with MASH, can enhance intestinal permeability and activate pro-inflammatory and pro-fibrotic pathways in hepatic cells, thereby contributing to liver dysfunction [104]. Additionally, lipopolysaccharide-positive (LPS+) bacterial EVs are more abundant in diet-induced obese (DIO) mice. These vesicles are linked to elevated expression of Toll-like receptor 4 (TLR4) and macrophage markers in the liver. They have the capability to enter the hepatic portal vein, subsequently reaching the liver, and can also escape the liver to enter the peripheral circulation [105]. This research not only enhances the understanding of microbial communication with distant organs but also introduces new possibilities for therapeutic interventions targeting EVs in metabolic and inflammatory diseases.

Furthermore, gut microbiota-derived EVs may exert beneficial effects in HFD-induced liver injury [106]. Additionally, the HLF/PPARα axis has been recognized as a pivotal mediator in mitigating MASLD by modulating gut microbiota-derived EVs, which in turn inhibit hepatocyte ferroptosis. The influence of the HLF/PPARα axis on these gut microbiota-derived EVs is further corroborated by the involvement of specific metabolites, such as taurochenodeoxycholic acid (TCDCA), a conjugated bile acid present within these EVs. This emerging therapeutic pathway underscores the complex interaction between intestinal factors and liver health, highlighting the potential of targeting gut-derived components to ameliorate liver disease [107].

Notably, the interaction between colorectal cancer (CRC)-derived EVs and liver-resident cells, such as HSCs and macrophages, plays a significant role in pre-metastatic niche (PMN) formation. For instance, exosomal miR-188-3p from CRC cells activates HSCs, promoting the establishment of a PMN through the AKT/mTOR pathway. This activation is crucial for liver metastasis and underscores the importance of EV-mediated communication in the metastatic process [108]. Similarly, exosomal miR-106a-5p induces M2 macrophage polarization, enhancing CRC liver metastasis by activating the JAK2/STAT3 pathway [109]. The clinical significance of these EV-mediated pathways is further supported by the detection of elevated levels of exosomal miR-188-3p and miR-106a-5p in the circulation of CRC patients. Their expression levels correlate with disease progression and liver metastasis, thus linking mechanistic insights with human pathological evidence.

Conversely, hepatocyte-derived EVs have been shown to enhance CRC liver metastasis in the context of fatty liver. This is facilitated by the upregulation of Rab27a expression in fatty liver, which increases EVs production. These EVs transfer Yes-associated protein (YAP)-regulating miRNAs to cancer cells, augmenting YAP activity and promoting an immunosuppressive environment through M2 macrophage infiltration [110]. Golgi protein 73 (GP73), a protein upregulated in hepatocytes, has been shown to modulate cholesterol content in endosomal compartments, thereby promoting exosome production. These exosomes, enriched with specific proteins such as neuron navigator 2 (NAV2), facilitate CRC liver metastasis, suggesting a novel therapeutic target in GP73 blockade. The clinical data highlights the translational significance of the GP73-NAV2 axis, revealing a positive correlation between serum GP73 and exosomal NAV2 levels in CRC patients with liver metastasis [111]. These mechanisms align with broader research on exosome-mediated cancer metastasis, where exosomes serve as critical vehicles for intercellular communication and metastatic niche formation.

### 4.3. Liver–Cardiovascular Axis

Emerging clinical evidence has increasingly underscored the substantial association between MASLD and CVD. MASLD is acknowledged as a multisystem disorder that not only compromises hepatic health but also contributes to the pathogenesis of cardiovascular complications [2,112,113]. The study of hepatic EVs in MAFLD provides insights into the complex interplay between liver-derived factors and vascular health [114,115,116]. For example, the novel-miRNA-7 identified in hepatic EVs targets lysosomal associated membrane protein 1 (LAMP1), promoting lysosomal membrane permeability and subsequent activation of the NLRP3 inflammasome, which is a critical mediator of endothelial hyperpermeability [117]. Another study has identified that hepatocyte-derived EVs containing miR-1 play a crucial role in promoting endothelial inflammation and atherogenesis. This finding is corroborated by observations indicating that steatotic hepatocytes release an increased quantity of EVs with altered microRNA profiles, which subsequently activate proinflammatory pathways in endothelial cells, resulting in elevated NF-κB activity and diminished expression of Kruppel-like factor 4 (KLF4) [42]. Supporting this central thesis, the study by Zeng et al. provides additional insights into the role of EVs in vascular complications associated with hepatic steatosis. It demonstrates that steatotic hepatocytes release EVs containing lectin galactoside-binding soluble 3 binding protein (Lgals3bp) that can reach vascular tissues and induce osteogenic differentiation in vascular smooth muscle cells, thereby accelerating vascular calcification [118]. Moreover, EVs from steatotic hepatocytes inhibit cholesterol efflux in macrophages, promoting foam cell formation and atherosclerosis progression via the miR-30a-3p/ABCA1 axis. Importantly, this mechanistic insight is corroborated by clinical observations, wherein serum small EVs from patients with MASLD, coupled with elevated expression of miR-30a-3p, are associated with reduced cholesterol efflux capacity in foam cells [116]. This finding aligns with the notion that hepatic steatosis can exacerbate vascular pathologies through EV-mediated pathways.

Collectively, these studies provide a comprehensive understanding of how hepatic steatosis and associated EV release contribute to inflammatory and vascular complications. The integration of these findings enhances the understanding of the complex interplay between liver pathology and vascular injury, emphasizing the critical role of EVs as mediators of disease progression. Moreover, the findings of hepatic-cardiovascular axis suggest that certain hepatic-derived EVs in the circulation can be used as early markers to predict cardiovascular events in patients with MASLD.

### 4.4. Liver–Pancreas Axis

Further elucidating the role of hepatocyte-derived EVs, another study demonstrates that these vesicles can induce inflammation in pancreatic islet macrophages via TLR4 signaling. This inflammatory cascade not only affects the liver but also extends to the pancreas, impairing beta cell function and insulin secretion, which are critical components in the metabolic dysfunction observed in MASLD. The study underscores the systemic impact of hepatocyte-derived EVs, highlighting their role in linking liver inflammation with broader metabolic disturbances [14]. Another study identifies miR-126a-3p as a critical factor in this process, where its upregulation in EVs from steatotic hepatocytes leads to pancreatic β-cell apoptosis and dysfunction [119]. This mechanism underscores the pathogenic link between MASLD and diabetes, suggesting potential therapeutic targets for intervention. Collectively, these studies underscore the critical role of EVs in mediating intercellular communication and disease progression in metabolic disorders. By targeting these EV-mediated pathways, it may be possible to mitigate the progression of diseases like diabetes and MASLD, highlighting the potential of EVs as both biomarkers and therapeutic targets in metabolic disease management.

### 4.5. Liver–Muscle Axis

Exercise-induced EVs have been demonstrated to play a pivotal role in systemic adaptations to physical activity by mediating inter-tissue signaling and contributing to the beneficial effects of exercise on health and disease prevention [120,121]. Recent research indicates that exercise in healthy humans prompts the release of EVs containing proteins capable of localizing in the liver and transferring their cargo, thereby suggesting a novel paradigm for exercise-induced systemic effects [121]. Additionally, another study investigates the potential of remote limb ischemic conditioning (RIC) as a therapeutic intervention for MASH. This study emphasizes the role of small EVs in facilitating muscle-to-liver communication, which is essential for mitigating steatohepatitis. The increased presence of miR-181d-5p in the liver, enabled by these vesicles, highlights the molecular advantages of RIC, particularly through the downregulation of nuclear receptor 4A3 (NR4A3). Additionally, translational evidence for this pathway is provided by human studies demonstrating that circulating EVs, isolated from individuals subjected to RIC, have been shown to ameliorate steatohepatitis and reverse related transcriptomic disturbances in both primary human hepatocytes and animal models. This underscores the conserved therapeutic potential of EV-mediated interorgan signaling [122].

In addition to muscle-to-liver communication, liver-derived EVs serve as a crucial nexus between hepatic pathology and muscular injury [123]. Notably, the development of a reactive oxygen species (ROS)-responsive manganese dioxide (MnO2) mesoporous hydrogel to modulate liver–muscle crosstalk and alleviate MASLD-associated sarcopenia through the delivery of exosomal miR-582-5p emerges as a promising therapeutic approach. This study underscores the hydrogel’s efficacy in reducing oxidative stress and inflammation, thereby preserving the integrity of muscle fibers and downregulating atrogenic markers in a mouse model of MASLD induced by a HFD [124].

## 5. EVs as Double-Edged Swords: Diagnostic and Therapeutic Implications

### 5.1. EVs as Biomarkers

In the context of MASLD, EVs have gained increasing recognition for their involvement in disease progression and their potential as biomarkers for diagnosis and prognosis [15,17,125]. The application of EVs in MASLD is particularly promising due to the demand for non-invasive diagnostic tools and the limitations associated with current invasive procedures, such as liver biopsies [126].

Research indicates that the proteomic profiles of circulating EVs can offer valuable insights into disease severity and progression, thereby providing a non-invasive diagnostic tool for MASH [127] (Table 1). Furthermore, elevated circulating levels of EpCAM+ CD133+ EVs have been observed in individuals with steatohepatitis compared to those with simple steatosis, highlighting their potential as biomarkers for the transition from simple steatosis to steatohepatitis [128]. Specifically, hepatocyte-derived EVs have been shown to correlate with steatosis and inflammation in patients with MASLD and MASH, suggesting their utility in monitoring disease progression and response to therapeutic interventions, such as weight loss surgery. The dynamic nature of EVs, which decrease following the resolution of MASLD due to weight loss surgery, further underscores their role as biomarkers [129]. Another study has demonstrated that the proportion of hepatogenic exosomes expressing glucose transporter 1 (GLUT1) is significantly elevated in patients with MASH compared to those with simple steatosis. This finding suggests that exosomal GLUT1 may serve as a molecular biomarker for the early detection and staging of liver fibrosis in MASLD [130]. Furthermore, the quantity of leukocyte-derived EVs is inversely correlated with the severity of fibrosis in MASLD [131]. Notably, plasma and subcutaneous abdominal adipose tissue (SAAT)-derived exosomes from individuals with obesity and MASLD decrease insulin signaling in myotubes and hepatocytes [85]. This study underscores the importance of exosome-mediated signaling in the systemic IR observed in MASLD. Recent advancements in machine learning and artificial intelligence have further enhanced the potential of EVs as biomarkers. By analyzing the size and concentration of circulating plasma EVs, alongside clinical and anthropomorphic data, researchers have developed models that can accurately stage MASLD and identify severe steatosis [126]. This approach not only highlights the diagnostic potential of EVs but also underscores the importance of integrating advanced computational techniques in the development of non-invasive diagnostic tools for liver diseases. Moreover, the proteomic analysis of serum EVs has identified specific proteins, such as Fibulin-3, as biomarkers that correlate with fibrosis progression and predict liver-related events in MASLD patients, highlighting the potential of EVs in clinical applications [16].

Recent investigations into the role of EVs in miRNA transport have revealed specific miRNA profiles, including miR-574-3p, miR-542-3p, and miR-200a-3p, in serum EVs that are associated with MASLD, thus providing an additional diagnostic dimension [132]. Additionally, circulating levels of let-7d-5p in plasma EVs are inversely associated with MASLD, indicating its potential as a blood-based biomarker for diagnosis [133]. Further studies have demonstrated that miRNAs derived from ASGR1+ EVs, such as miR-122, miR-192, and miR-128-3p, which are liver-specific, exhibit a strong correlation with disease severity, offering a promising alternative for the non-invasive diagnosis and monitoring of MASLD [134].

In addition to examining blood-derived EVs, the lipidomic profiles of urinary EVs, including free fatty acids (FFA) such as FFA (18:0) and FFA (18:1), lysophosphatidylcholine (LPC) (22:6/0:0), and phosphatidylinositol (PI) (16:0/18:1), were characterized. This characterization demonstrated their potential to distinguish MASH from simple steatosis. The integration of lipidomic data with machine learning techniques, as employed in the urinary EV study, substantially enhances the diagnostic accuracy for MASH, achieving an area under the curve (AUC) of 92.3% for the identified lipid biomarker panel [135].

Recently, a study by Li et al. introduces an innovative method for diagnosing and monitoring MASLD utilizing a near-infrared fluorescent probe specifically targeting EVs. This cutting-edge approach effectively addresses the challenges inherent in the in situ detection of EVs, which are progressively acknowledged as significant biomarkers for a range of diseases, including MASLD. The probe, designated ND-N, is engineered to amplify fluorescence in response to elevated viscosity, a hallmark of the MASLD microenvironment, thus enabling real-time imaging of EVs in vivo [136].

### 5.2. Therapeutic Strategies Targeting EVs

In addition to their diagnostic potential, EVs represent promising targets for therapeutic interventions in MASLD. For example, interleukin-22 (IL-22) has been demonstrated to inhibit the inflammatory functions of hepatocyte-derived EVs, thereby attenuating liver inflammation in MASH [45]. Moreover, nuciferine, an extract obtained from lotus leaves, has demonstrated efficacy in reducing hepatic inflammation associated with MASH. This effect is mediated through the modulation of the SIRT1/NF-κB signaling pathway and the enhancement of EVs derived from Akkermansia muciniphila [137]. Additionally, the therapeutic potential of total saponins from Panax japonicus (TSPJ) in MASLD appears promising. The primary mechanism by which TSPJ exerts its effects involves the upregulation of miR-463-5p in hepatic macrophage-derived exosomes, which subsequently leads to the downregulation of PHD2 expression in hepatocytes, thereby improving lipid metabolism [138]. A separate study highlights the efficacy of MSDC-0602, a next-generation thiazolidinedione, in mitigating liver fibrosis and stellate cell activation in a rodent model of MASH by inhibiting the release of EVs from hepatocytes [139]. Additionally, semaglutide, a glucagon-like peptide-1 receptor agonist, has shown promise in the treatment of MASLD, particularly in the context of T2DM. The research emphasizes semaglutide’s role in modulating ECM production in HSCs, which is facilitated through the action of exosomes [140]. Collectively, strategies to modulate the release of EVs might be developed for the treatment of patients with MASLD. By targeting these EVs with interfering RNA or antibodies, it is possible to inhibit their profibrotic or inflammatory effects through the suppression of specific molecules present on the EVs.

### 5.3. Therapeutic Applications of EVs

The therapeutic potential of EVs in MASLD is underscored by their ability to encapsulate and deliver a diverse array of bioactive molecules, including proteins, nucleic acids, and lipids, to target cells. This capability allows EVs to modulate gene expression and signaling pathways, thereby influencing cellular behavior and disease progression [141].

#### 5.3.1. Therapeutic EVs of Natural Origin

**Figure 4:**
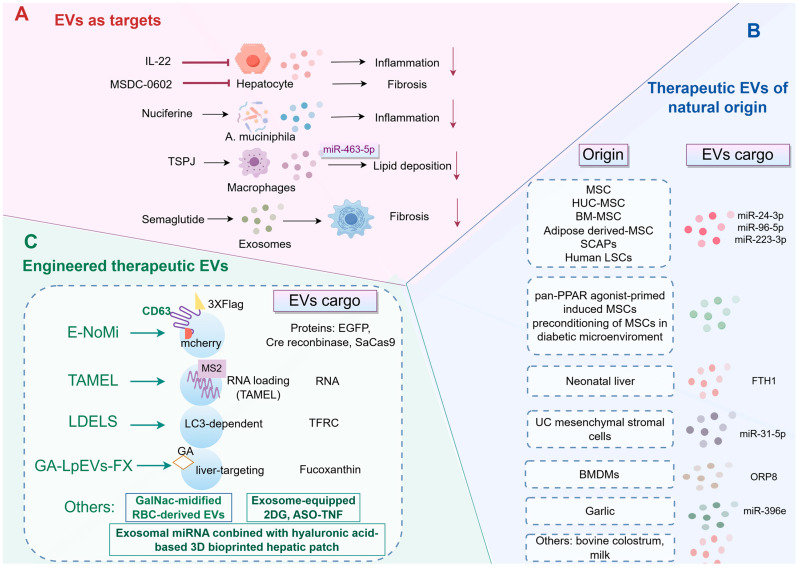
Schematic overview of the therapeutic landscape of EVs in MASLD. (**A**) EVs as therapeutic targets. Several pharmacological agents, such as IL-22, Semaglutide, and Nuciferine, achieve their therapeutic effects by modulating EV pathways. This modulation results in improvements in key pathological features of MASLD, including inflammation, fibrosis, and lipid deposition. (**B**) Therapeutic EVs of natural origin. A diverse array of native EVs, derived from various cellular sources such as stem cells from different tissues, liver cells, macrophages, and dietary sources, exhibit intrinsic therapeutic properties for the treatment of MASLD. (**C**) Strategies to enhance EV efficacy through engineering. BMDMs, bone marrow-derived macrophages; BM-MSC, bone marrow-derived mesenchymal stem cell; 2DG, 2-deoxy-D-glucose; EVs, extracellular vesicles; FTH1, ferritin heavy chain 1; GalNac, N-acetyl galactosamine; HUC, human umbilical cord; IL-22, interleukin-22; LDELS, LC3-dependent EV loading and secretion; LSCs, liver stem cells; MSCs, mesenchymal stem cells; ORP8, oxysterol-binding protein-related protein 8; RBC, red blood cell; SCAPs, stem cells of the apical papilla; TAMEL, Targeted and Modular EV Loading; TFRC, transferrin receptor; TNF, tumor necrosis factor; TSPJ, total saponins from Panax japonicus.

Recent research has underscored the potential of stem cell-derived EVs in mitigating key pathogenic pathways associated with MASLD (Figure 4). Mesenchymal stem cells (MSCs) derived exosomes containing RING finger protein 31 (RNF31) have demonstrated the ability to modulate mitochondrial fission and mitophagy. This modulation leads to improvements in mitochondrial dysfunction and hepatic steatosis, which are critical for the management of MASLD [18,142]. Specifically, MSC-derived EVs have been shown to influence AMPK signaling, inhibit the release of FFAs, and reduce CD36 expression, thereby ameliorating hepatic steatosis and facilitating liver regeneration [143]. The anti-inflammatory and antioxidative properties of exosomes derived from human umbilical cord (HUC)-MSCs have been further illustrated in studies demonstrating their capacity to ameliorate liver steatosis by enhancing fatty acid oxidation and decreasing fatty acid synthesis, thus improving lipid metabolism and reducing hepatic inflammation [144,145,146]. These findings align with the effects observed in exosomes derived from stem cells of the apical papilla (SCAPs), which have been shown to enhance hepatic fatty acid oxidation and suppress fatty acid synthesis, thereby alleviating hepatic fat accumulation and liver damage in models of MASH [147]. Additionally, EVs derived from human liver stem cells (LSCs) have been shown to exert anti-fibrotic and anti-inflammatory effects, leading to significant improvements in liver function and a reduction in fibrosis in murine models of MASH [148]. It is noteworthy that miRNAs play a crucial role in the stem cell therapy of MASLD. For instance, exosomes from MSCs have been shown to modulate macrophage polarization via the miR-24-3p/STING axis, thereby mitigating inflammation and fibrosis in MASH models [149]. Similarly, miR-96-5p, another component of bone marrow MSC (BM-MSC)-derived EVs, has been found to alleviate MASH by inhibiting caspase-2 signaling [150]. In addition, adipose-derived MSC-EVs have been reported to mitigate MASLD by delivering miR-223-3p, which targets and suppresses E2F1, consequently reducing lipid accumulation and liver fibrosis [151,152]. Notably, research by Kim et al. underscores the therapeutic potential of EVs derived from pan-PPAR agonist-primed induced MSCs in ameliorating MASH symptoms. These EVs exhibit distinct protein signatures that contribute to reducing steatotic changes and oxidative stress while promoting liver regeneration [153]. Moreover, the preconditioning of MSCs in a diabetic microenvironment appears to enhance the therapeutic efficacy of their derived exosomes. This preconditioning process increases the secretion capacity and anti-inflammatory activity of MSCs, thereby enhancing their ability to inhibit hepatocyte pyroptosis and ameliorate MASLD [154]. Despite promising preclinical outcomes, the clinical implementation of stem cell-derived EVs continues to encounter several challenges. These include the need for standardization in large-scale production, rigorous elimination of tumorigenic potential, and the possibility of immunogenic responses associated with allogeneic transplantation.

In addition to stem cells, EVs derived from UC mesenchymal stromal cells accumulate in the liver, where they deliver miR-31-5p to suppress platelet-derived growth factor B (PDGFB) produced by hepatic macrophages, thereby impeding the progression of MASLD and enhancing neurovascular health [155]. Another study demonstrates that these EVs can significantly mitigate body weight loss and liver damage in mice, primarily through the modulation of inflammatory cytokines and the restoration of PPARα protein expression in liver cells [156]. Recently, the study on neonatal liver-derived ferritin heavy chain 1 (FTH1)-enriched EVs presents a compelling argument for their role in attenuating ferroptosis and ameliorating MASLD pathogenesis [157]. Moreover, EVs originating from BMDMs have been demonstrated to influence hepatic inflammation and lipotoxicity, both of which are pivotal in the progression of MASH. This study identifies ORP8 as a critical component within these EVs, which plays a role in mitigating ER stress, thereby presenting a novel therapeutic target for MASH [75].

Recently, the exploration of plant-derived EVs as a therapeutic strategy for MASLD represents a promising frontier in medical research. The findings reveal that garlic-derived exosomes, through miR-396e, modulates PFKFB3 expression, thereby reducing inflammatory responses and enhancing lipid metabolism in hepatocytes [158]. Additionally, bovine colostrum-derived EVs could also protect against MASH by promoting the proliferation of Akkermansia and enhancing the production of tight junction proteins and mucin, reinforcing the intestinal barrier [159]. Similarly, the study of milk-derived EVs has garnered significant attention due to their potential role in maintaining intestinal barrier integrity, particularly within the gut–liver axis. These vesicles, derived from both bovine and human milk, have been shown to restore gut barrier integrity at multiple levels, underscoring their potential as therapeutic agents in managing gut-related disorders and associated MASH [160].

#### 5.3.2. Engineered Therapeutic EVs

One promising approach to improving EV cargo loading involves the engineering of specific proteins associated with EV membranes. For instance, the engineering of the CD63 transmembrane protein has been shown to facilitate selective cargo loading into EVs. By modifying CD63 to include a mCherry tag inside the EV membrane and a 3xFLAG tag on the outside, researchers were able to selectively enrich EVs with desired cargo proteins, such as EGFP and CRISPR-Cas nucleases. This engineered system, known as E-NoMi, demonstrated enhanced payload delivery, being ten times more effective than traditional methods like size-exclusion chromatography [161]. In addition to protein engineering, the development of platforms for actively loading RNA cargo into EVs has provided insights into the limiting steps of EV-mediated delivery. The Targeted and Modular EV Loading (TAMEL) approach, which involves the fusion of MS2 bacteriophage coat proteins to EV-associated proteins, has been shown to significantly enhance RNA loading into EVs. This method allows for a 40-fold enrichment of RNA cargo, particularly benefiting smaller RNA molecules. However, despite high loading efficiencies, the delivery of these cargos to recipient cells often encounters barriers such as rapid degradation, suggesting that inefficient endosomal fusion or escape may limit EV-mediated transfer [162]. Furthermore, the role of autophagy-related pathways in EV cargo loading has been explored, revealing that the LC3-dependent EV loading and secretion (LDELS) pathway facilitates the packaging of cytosolic cargos into EVs. This pathway involves components of the autophagy machinery and the ESCRT pathway, and it has been shown to promote the secretion of specific transmembrane proteins, such as the transferrin receptor (TFRC), via EVs [163].

The engineering of EVs to augment their therapeutic efficacy has recently emerged as a prominent focus of research. In a 2025 study, He et al. demonstrates that exosomes, when loaded with antisense oligodeoxynucleotide targeting tumor necrosis factor (TNF) or 2-deoxy-D-glucose (2DG), significantly suppress inflammatory mediators in hepatic macrophages, thereby ameliorating experimental steatohepatitis in murine MASH models induced by a choline deficient amino acid-defined diet. This intervention also upregulates superoxide dismutase 1 (Sod1), indicating a novel mechanism for reducing oxidative stress in MASH [164]. Another study introduces an innovative approach to treating MASLD by employing EVs as delivery vehicles for fucoxanthin. This method capitalizes on the biocompatibility and immunological properties of EVs, which are derived from Lactobacillus paracasei and modified with glycyrrhetinic acid to enhance liver-targeting capabilities. The findings demonstrate that these vesicles effectively reduce hepatic lipid accumulation and oxidative stress, presenting a promising therapeutic strategy for MASLD [165]. Furthermore, the incorporation of triantennary N-acetyl galactosamine (GalNac) sequences enhances the specificity of red blood cell (RBC)-derived EVs for hepatocytes, thereby improving therapeutic outcomes in models of MASLD when loaded with PJ34 [166]. More recently, Saha et al. have investigated a novel therapeutic approach for MASLD by employing exosomal miRNA, either alone or in combination with hepatocytes, in conjunction with a three-dimensional bioprinted hyaluronic acid-based hepatic patch. This innovative strategy aims to address the complex metabolic pathways involved in MASLD, which are often inadequately understood, by providing a sustained and localized release of therapeutic agents. The use of MSC-derived exosomes is particularly promising due to their capacity to modulate various cellular processes, including inflammation and fibrosis, which are central to the pathogenesis of MASLD [167].

Collectively, by modifying the surface and cargo of EVs, it is feasible to enhance their targeting capabilities and efficacy in delivering anti-inflammatory or anti-fibrotic agents directly to the liver. This strategy has the potential to reduce the systemic side effects commonly associated with conventional therapies and offers a more precise treatment modality for MASLD. However, the clinical application of EV-based therapies encounters several challenges, including potential off-target effects, immunogenicity, and concerns regarding long-term safety. Furthermore, the risk of tumorigenicity associated with stem cell-derived EVs underscores the need for stringent purification and thorough characterization processes.

### 5.4. Clinical Trials of EVs in Liver Diseases

The progression of EV research from laboratory investigations to clinical applications is demonstrated by an increasing number of clinical trials, which we have systematically categorized in Table 2 (https://clinicaltrials.gov/ accessed on 13 January 2026). These studies collectively delineate the translational trajectory of EVs in the context of liver diseases: from their role as diagnostic and prognostic biomarkers in conditions such as NAFLD and cirrhosis, to their function as pharmacodynamic indicators of drug response, and ultimately to their evaluation as innovative therapeutic agents. This includes approaches such as MSC-derived EV infusions and engineered exosome-based pharmaceuticals. Notably, mechanistic investigations, such as those in study NCT04758793, explore EV-mediated liver–muscle interactions in sarcopenia, providing essential insights that underpin these clinical applications. The advancement of these trials will be crucial in substantiating the therapeutic potential of EVs and addressing the practical challenges associated with scale-up and regulatory compliance, which are discussed in subsequent sections.

## 6. Challenges and Future Perspectives

### 6.1. Standardization and Reproducibility

A significant challenge in EVs research is the absence of standardized methodologies for their isolation, characterization, and functional analysis. The heterogeneity of biofluids and the diversity of EV subpopulations result in techniques such as differential ultracentrifugation, size-exclusion chromatography, and polymer-based precipitation producing markedly different EV populations with unique cargo profiles [168]. This variability compromises the reproducibility of findings across laboratories and complicates the comparative analysis of clinical data. Future endeavors must rigorously adhere to and expand upon the Minimal Information for Studies of Extracellular Vesicles (MISEV) guidelines [169]. The development of robust, high-throughput, and clinically applicable isolation protocols is essential for the reliable identification and validation of EV-based biomarkers and for ensuring the consistent quality of therapeutic EV preparations.

### 6.2. Understanding In Vivo Kinetics and Targeting

The therapeutic application of EVs, whether as intrinsic biological agents or engineered drug delivery systems, encounters substantial translational challenges associated with their in vivo behavior. Essential questions regarding their pharmacokinetics, biodistribution, tissue tropism, and potential immunogenicity remain insufficiently addressed [170]. Following systemic administration, most EVs are predominantly sequestered by the liver and spleen. However, achieving precise targeting to specific hepatic cell types, such as activated HSCs versus hepatocytes, remains a significant obstacle. Future research should prioritize the rational engineering of EV surfaces to enhance organotropic and cell-specific targeting, such as by incorporating ligands for receptors that are overexpressed on target cells [171]. Simultaneously, gaining a more comprehensive understanding of the natural homing signals that regulate EV distribution in vivo will be essential for optimizing both native and engineered EV therapies.

Significant interspecies differences between human MASLD and commonly utilized murine models may profoundly affect the biological understanding and translational interpretation of EVs research. These disparities are evident across multiple dimensions. Firstly, the timeline and progression dynamics of the disease are fundamentally distinct. Human MASLD develops over several decades, influenced by a complex interplay of genetic predisposition, diet, lifestyle, and gut microbiome factors. In contrast, murine models, whether induced by a high-fat diet, a methionine-choline-deficient diet, or genetic modifications such as *ob*/*ob* mice, achieve steatosis, inflammation, and fibrosis within a matter of weeks to months. This accelerated timeline may alter the kinetics of EVs release, bias the cargo composition towards acute stress signals, and fail to replicate the chronic, low-grade EV-mediated communication characteristic of the human condition. Secondly, inherent biological differences in metabolism, immune system function, and liver cell biology exist between mice and humans. For example, murine macrophages and HSCss may respond differently to EVs cargo compared to their human counterparts. Additionally, the miRNA repertoire carried by EVs exhibits species specificity. This implies that a pro-fibrotic miRNA identified in mouse EVs may not be applicable or similarly regulated in human MASLD. Such interspecies variations significantly impact the translational applicability of EV research. An EV-based biomarker deemed promising in mouse plasma might not exhibit equivalent sensitivity or specificity in human serum, due to distinct baseline profiles and the presence of confounding comorbidities in patients. Likewise, therapeutic EVs engineered or selected for efficacy in mouse models may lack effectiveness or produce off-target effects in humans, attributable to differences in EV tropism and the biology of recipient cells.

### 6.3. Integration of Multi-Omics and Advanced Technologies

The combination of single-cell RNA sequencing with EV secretion analysis holds promise for identifying specific cellular subpopulations that are predominant or pathogenic producers of EVs in diseased liver tissue. Furthermore, the application of spatial transcriptomics and imaging mass cytometry, alongside in situ EV detection techniques, can illuminate the spatial distribution of EV release and uptake within the liver’s architecture, thereby revealing previously unrecognized communication niches. Additionally, the use of artificial intelligence and machine learning on integrated multi-omics datasets, including proteomic, lipidomic, and transcriptomic data from circulating EVs, offers the potential to elucidate their cellular origins and identify highly predictive, multi-component biomarker signatures [126]. This approach is expected to enhance the precision of disease staging and prognostication.

### 6.4. Navigating the Path to Clinical Translation

The clinical translation of EV-based therapeutics encounters substantial challenges in both regulatory and manufacturing domains. A central issue is the ambiguous regulatory classification of EVs, which can be categorized as biologics, advanced therapy medicinal products, or medical devices, contingent upon the jurisdiction. This regulatory uncertainty complicates the selection of approval pathways and the design of pivotal clinical trials. Furthermore, the absence of standardized, scalable manufacturing processes constitutes a significant impediment to commercialization. Transitioning from laboratory-scale isolation to large-scale production necessitates methods that effectively balance yield, purity, and EV functionality. Ensuring batch-to-batch consistency in EV yield, purity, composition, and bioactivity is particularly challenging due to biological variability in source cells and EV heterogeneity. Consequently, it is imperative to define critical quality attributes, establish robust potency assays, and exclude potentially oncogenic or immunostimulatory components.

In addition to production challenges, the establishment of sustainable business models for EV therapies remains uncertain. A critical issue pertains to the identification of stakeholders responsible for financing a complex and potentially costly biologic treatment for chronic conditions such as MASLD. It is imperative to demonstrate not only the efficacy but also the cost-effectiveness of these therapies to facilitate their adoption in clinical practice.

Looking ahead, the field must advance on several interconnected fronts. From a technological perspective, the integration of single-cell omics with EVs analysis will facilitate the association of EV signatures with their cellular origins and functional states. Advanced engineering strategies, such as the development of EVs that selectively release their cargo in diseased tissues, could improve targeting and safety. Equally crucial is the need for early and sustained collaboration among researchers, clinicians, industry stakeholders, and regulatory bodies to establish common standards, design clinically meaningful trials, and develop a well-defined translational roadmap.

In conclusion, the effective clinical application of EV-based therapies necessitates concurrent advancements in standardized manufacturing processes, the establishment of more precise regulatory frameworks, and comprehensive biological characterization. By addressing these interconnected challenges, the field can progress from merely elucidating EV biology to fully leveraging its potential for concrete patient benefits in MASLD and other medical conditions.

## 7. Conclusions

This review underscores three principal conceptual advancements: (1) the characterization of MASLD as a systemic communication disorder facilitated by EVs, (2) the elucidation of the dual function of EVs as both contributors to pathology and potential therapeutic agents, and (3) the incorporation of multi-omics and engineering methodologies to propel EV-based precision medicine forward.

While EVs are key in MASLD communication, challenges remain in standardizing EV separation and characterizing biofluids for molecular diagnostics. Additionally, identifying unique cargo traits for specific liver disease stages is crucial for future personalized medicine. There are several unresolved questions regarding the use of EVs as therapeutics strategies, such as their kinetics, toxicity, off-target effects and the delivery to precise types of cells. By overcoming these obstacles through collaborative, rigorous, and innovative scientific endeavors, the field is well-positioned to realize the full potential of EVs, transitioning them from intriguing biological entities into effective tools for the management and treatment of MASLD.

## Figures and Tables

**Table 1:** EVs as biomarker in MASLD.

EVs Biomarker	Origin	Predictive Role in MASLD	Reference
Proteomic signatures	Serum	MASH	[127]
EpCAM+ CD133+ EVs	Serum	simple steatosis to steatohepatitis	[128]
EV counts; sphingolipids	Plasma, hepatocyte	MASLD resolution due to weight loss surgery	[129]
GLUT1	Plasma, hepatocyte	simple steatosis to steatohepatitis and fibrosis	[130]
CD14+ and CD16+ EV	Plasma, leukocyte	Predict liver fibrosis severity	[131]
Exosomes	Plasma and SAAT	Systemic IR	[85]
EV size and concentration	Plasma	Stage MASLD and identify severe steatosis	[126]
Fibulin-3	Serum	Predict liver-related events	[16]
miR-574-3p, miR-542-3p, and miR-200a-3p	Serum	MASLD	[132]
let-7d-5p	Plasma	MASLD	[133]
miR-122, miR-192, and miR-128-3p	Plasma, liver-specific	Distinguish patients with MAFL and MASH	[134]
FFA (18:0), LPC (22:6/0:0), FFA (18:1), and PI (16:0/18:1)	Urine	MASH	[135]
Fluorescent probe imaging EVs	Liver	MASLD	[136]

**Table 2:** Clinical trials involving EVs with relevance to liver diseases.

ID	Phase/Status	Primary Population	EV Role & Key Measurement
NCT06825572	Phase 1; not yet recruiting	Patients with acute-on-chronic liver failure undergoing first liver transplantation	Therapeutic agent; safety and tolerability of a single intravenous infusion of allogeneic MSC-EVs post-transplantation
NCT05940610	Phase 1/2; withdrawn	Patients with acute or acute-on-chronic liver failure (non-transplant)	Therapeutic agent; safety and efficacy of multiple intravenous infusions of allogeneic MSC-EVs as an adjunct to standard medical therapy
NCT05881668	Phase 1; withdrawn	Patients with acute-on-chronic liver failure after liver transplantation	Therapeutic agent; safety and tolerability of a single postoperative intravenous infusion of allogeneic MSC-EVs
NCT05871463	Phase 2; last known: recruiting	Patients with decompensated liver cirrhosis	Therapeutic agent; safety and efficacy evaluation of umbilical cord MSC-derived exosomes as an alternative to cell therapy
NCT07131306	Phase 2; not yet recruiting	Non-viral acute on chronic liver failure patients receiving regenerative therapy	Biomarker of therapeutic efficacy; EVs quantity monitored as an immunomodulatory marker to assess response to MSC therapy
NCT05375604	Phase 1; terminated	Patients with advanced HCC or liver metastases from gastric/CRC	Therapeutic agent; safety and efficacy evaluation of an engineered exosome loaded with an antisense oligonucleotide targeting STAT6
NCT04191044	N/A; not yet recruiting	NAFLD patients stratified by disease severity	EVs in liquid biopsy as exploratory diagnostic biomarkers for portal hypertension
NCT06236568	N/A; recruiting	HCC patients within Milan criteria undergoing liver transplantation	Prognostic biomarker in liquid biopsy; exosome/miRNA analysis to predict HCC recurrence and ischemia–reperfusion injury
NCT06396871	N/A; recruiting	Adults stratified by metabolic disease states	EVs analyzed as one component of circulating factors to characterize differences across metabolic diseases
NCT06814990	N/A; recruiting	Patients with decompensated alcohol-related cirrhosis undergoing transjugular intrahepatic portosystemic shunt (TIPSS)	Bacterial EVs analyzed as part of a comprehensive marker set to understand post-TIPSS cardiac decompensation
NCT06687265	Phase 2; recruiting	Cirrhosis patients with portal hypertension on carvedilol	Pharmacodynamic biomarker; hepatocyte large EVs as a marker of hepatocyte stress to evaluate metformin’s effect
NCT04250259	Phase 2; recruiting	Patients with alcoholic cirrhosis	Biomarker of treatment response; measurement of CYP2E1-enriched microparticles in blood as a marker of cellular oxidative/ER stress to assess the effect of S-adenosylmethionine
NCT07185360	N/A; not yet recruiting	Compensated cirrhosis	Circulating EVs as prognostic biomarkers; longitudinal protein/size profiling
NCT05699863	N/A; active, not recruiting	Obese patients with NAFLD undergoing lifestyle intervention	EV concentration/phenotype (flow cytometry) as diagnostic/monitoring biomarkers
NCT01104220	N/A; active, not recruiting	Adults stratified by weight and metabolic status	Mechanistic signal in inter-organ crosstalk; exosomes from blood and adipose tissue as mediators in obesity-related inflammation
NCT03701828	N/A; completed	Obese patients with NAFLD scheduled for bariatric surgery	Analysis of EV quantity, content, and function from adipose, liver, and blood tissues before and after ~20% weight loss
NCT04758793	N/A; not yet recruiting	Adult patients undergoing scheduled abdominal surgery, stratified by liver disease stage	Pathological mediators; profiling of circulating liver or muscle-derived EVs

## Data Availability

No new data were created or analyzed in this study. Data sharing is not applicable to this article.

## Abbreviations

ADAMTS1, a disintegrin and metalloproteinase with thrombospondin motifs 1; AKR1B7, aldo-keto reductase 1B7; APOE, apolipoprotein E; ATMs, adipose tissue macrophages; AUC, under the curve; BMDMs, bone marrow-derived macrophages; BMI, body mass index; CKD, chronic kidney disease; CRC, colorectal cancer; CVD, cardiovascular disease; CXCL10, C-X-C motif ligand 10; 2DG, 2-deoxy-D-glucose; DIO, diet-induced obese; ECs, endothelial cells; ECM, extracellular matrix; ER, endoplasmic reticulum; ESCRT, Endosomal Sorting Complex Required for Transport; EVs, extracellular vesicles; FFAs, free fatty acids; FTH1, ferritin heavy chain 1; GalNac, N-acetyl galactosamine; Ggpps, geranylgeranyl diphosphate synthase; GLUT1, glucose transporter 1; GP73, Golgi protein 73; HCC, hepatocellular carcinoma; HFD, high-fat diet; HSCs, hepatic stellate cells; HUC, human umbilical cord; IGFBP7, insulin-like growth factor-binding protein 7; IL6, interleukin 6; IL-22, interleukin-22; ILVs, intraluminal vesicles; IR, insulin resistance; IRE1A, endoplasmic reticulum to nucleus signaling 1; KCs, Kupffer cells; KLF4, Kruppel-like factor 4; LDELS, LC3-dependent EV loading and secretion; LDLR, low-density lipoprotein receptor; LAMP1, lysosomal associated membrane protein 1; Lgals3bp, lectin galactoside-binding soluble 3 binding protein; LIMA1, LIM domain and actin binding 1; LPC, lysophosphatidylcholine; LPS+, lipopolysaccharide-positive; LSCs, liver stem cells; LSECs, liver sinusoidal endothelial cells; MASH, metabolic dysfunction-associated steatohepatitis; MASLD, metabolic dysfunction-associated steatotic liver disease; MASP1, mannan-binding lectin serine protease 1; miRNAs, microRNAs; MISEV, Minimal Information for Studies of Extracellular Vesicles; MLK3, mixed lineage kinase 3; MnO2, manganese dioxide; MSCs, mesenchymal stem cells; mtDNA, mitochondrial DNA; MVBs, multivesicular bodies; NAFLD, nonalcoholic fatty liver disease; NAV2, neuron navigator 2; NETs, neutrophil extracellular traps; NR4A3, nuclear receptor 4A3; ORP8, oxysterol-binding protein-related protein 8; OSA, obstructive sleep apnoea; PA, palmitic acid; PDGFB, platelet-derived growth factor B; PI, phosphatidylinositol; PMN, pre-metastatic niche; PUM1, pumilio1; RBC, red blood cell; RBP4, retinol-binding protein 4; RIC, remote limb ischemic conditioning; RNF31, RING finger protein 31; ROCK1, Rho-associated protein kinase 1; ROS, reactive oxygen species; S1P, sphingosine-1-phosphate; SAAT, subcutaneous abdominal adipose tissue; SCAPs, stem cells of the apical papilla; Sod1, superoxide dismutase 1; T2DM, type 2 diabetes mellitus; TAMEL, Targeted and Modular EV Loading; TAZ, transcriptional co-activator with PDZ-binding motif; TCDCA, taurochenodeoxycholic acid; TFRC, transferrin receptor; TGFBR1, transforming growth factor beta receptor 1; Th17, T-helper 17; TLR4, Toll-like receptor 4; TIPSS, transjugular intrahepatic portosystemic shunt; TNF, tumor necrosis factor; TRAIL, tumor necrosis factor-related apoptosis-inducing ligand; Tregs, regulatory T cells; TSPJ, total saponins from Panax japonicus; VAT, visceral adipose tissue; YAP, Yes-associated protein.
